# Integrating high-fidelity hiPSC-cardiomyocytes with AI-driven modeling for enhanced proarrhythmic risk assessment

**DOI:** 10.1007/s00204-026-04361-8

**Published:** 2026-04-28

**Authors:** Su-Bin Kim, Jaehun Lee, Jieun An, Ara Cho, Kun Hee Lee, Hwan Choi, Choongseong Han, Muhammad Adnan Pramudito, Ki Moo Lim, Dong-Hun Woo

**Affiliations:** 1 Department of Commercializing iPSC Technology, NEXEL Co., Ltd., 8th floor, 55 Magokdong-Ro, Gangseo-Gu, Seoul, 07802 Republic of Korea; 2 Department of Biomedical Engineering, Kumoh National Institute of Technology, Gumi, 39177 Republic of Korea; 3 Department of IT Convergence Engineering, Kumoh National Institute of Technology, Gumi, 39177 Republic of Korea; 4 Meta Heart Co., Ltd., Gumi, 39177 Republic of Korea

**Keywords:** Human induced pluripotent stem cell-derived cardiomyocytes, Cardiotoxicity, AI-integrating model, Drug screening, Anticancer drugs

## Abstract

Cardiotoxicity remains the leading driver of drug attrition; however, its prediction remains suboptimal when conventional hERG assays and animal models are used. Human induced pluripotent stem cell-derived cardiomyocytes (hiPSC-CMs) offer a human-relevant alternative that aligns with the CiPA initiative and ICH E14/S7B guidelines. This study validated an integrated platform that combines high-purity hiPSC-CMs with Artificial Intelligence (AI) to enhance the accuracy of predicting drug-induced proarrhythmic risk. Phenotypic characterization of the hiPSC-CMs demonstrated high cardiac differentiation efficiency (cTnT +  > 95%) and a predominant ventricular-like identity (MLC-2 V + , 78–84%), ensuring biological relevance for ventricular arrhythmia assessment. Electrophysiological data from 28 CiPA reference compounds were collected via Multielectrode Array (MEA) to train multiple machine learning models. The Artificial Neural Network outperformed the other architectures, achieving a superior ROC-AUC of 0.982. The utility of the platform was evaluated using 12 anticancer agents. Although most drugs showed dose-dependent reductions in impedance-based viability, four compounds (Idarubicin, Erlotinib, Sunitinib, Cyclophosphamide) did not exhibit overt structural cytotoxicity. However, MEA analysis revealed significant functional perturbations, including FPDcF prolongation, in sunitinib- and erlotinib-treated samples after long-term treatment. The AI model subsequently classified these two agents as high-to-intermediate risk for Torsades de Pointes (TdP), thereby quantifying their time-dependent proarrhythmic liabilities. These findings show the platform’s ability to detect hidden functional cardiotoxicity, often missed by standard viability assays. The AI-hiPSC-CM system offers a high-throughput, early-stage safety screening tool that bridges in vitro data and clinical outcomes with a standardized risk assessment framework.

**Supplementary Information:**

The online version contains supplementary material available at 10.1007/s00204-026-04361-8.

## Introduction

Drug-induced cardiotoxicity remains a formidable challenge in the pharmaceutical industry (Jiang et al. 2016; Li et al. 2017, 2020), frequently leading to late-stage attrition in clinical trials or to post-market withdrawal of approved therapeutics (Blinova et al. 2018). Historically, preclinical safety assessments have focused on blocking of the human ether-à-go-go-related gene (hERG) potassium channel as a surrogate for life-threatening ventricular arrhythmia, such as Torsades de Pointes (TdP) (Gintant 2008). However, the hERG-centric paradigm and non-human in vivo telemetry often lack sufficient predictive accuracy. This insufficiency is largely attributed to the complex, integrated nature of cardiac repolarization, which is governed by multiple interacting ion channels, and inherent species-specific differences in electrophysiology, limiting the translatability of animal models to human clinical outcomes (Hamlin and Keene 2020).

The recent enactment of the FDA Modernization Act 2.0 has further catalyzed a global shift away from mandatory animal testing, placing a premium on the development of validated New Approach Methodologies (NAMs). Within this regulatory landscape, the Comprehensive in Vitro Proarrhythmia Assay (CiPA) initiative was established, proposing a mechanistic framework that integrates multiple cardiac ion channel assays, in silico modeling, and functional assessment in human-relevant cellular models (Strauss et al. 2019). Human induced pluripotent stem cell-derived cardiomyocytes (hiPSC-CMs) have emerged as cornerstones of this framework (Sharma et al. 2013). Unlike single-ion channel assays, hiPSC-CMs provide a comprehensive electrophysiological environment that closely mimics the human cardiac tissue, thus facilitating a more holistic evaluation of drug effects (Harris et al. 2013). While the utility of hiPSC-CMs has been validated using CiPA 28 reference compounds, there remains a critical need for rigorous phenotypic characterization, particularly to achieve high-purity, ventricular-like populations, to ensure the consistency and reliability of safety signals across laboratories (Sala et al. 2017; Lee et al. 2024). Given that TdP is fundamentally a ventricular phenomenon characterized by delayed repolarization and early after-depolarization, the use of subtype-specific cells with high biological fidelity is essential to capture nuanced electrophysiological perturbations relevant to clinical outcomes.

Although hiPSC-CMs coupled with MEA technology generate high-content multiparameter datasets, interpreting these complex signals through traditional threshold-based analysis remains a bottleneck (Guerrelli et al. 2024). The integration of Artificial Intelligence (AI) and Machine Learning (ML) offers a transformative solution. AI-driven models can discern subtle, non-linear relationships between multi-parametric electrophysiological changes and clinical proarrhythmic risk, thereby significantly enhancing the predictive power of in vitro assays (Golgooni et al. 2019; Serrano et al. 2023). Several groups have begun applying machine learning to MEA recordings from hiPSC‑CMs, primarily using limited feature sets or a single modeling strategy. For example, Blinova et al. combined field potential duration corrected by Fridericia’s formula (FPDcF) changes with logistic regression to classify the TdP risk in CiPA compounds, whereas Serrano et al. used handcrafted time‑series features with linear models to improve prediction performance (Blinova et al. 2018; Serrano et al. 2023). A recent study by Pramudito et al. used a stacking ensemble of random forest, XGBoost, and a shallow Artificial Neural Network (ANN) trained on two ΔFPDcF‑derived predictors, achieving excellent discrimination on CiPA MEA data Park et al. 2025). Yun‑Gwi Park et al. further extended MEA‑based modeling to disease‑specific and patient‑derived hiPSC‑CMs using ANN, Random Forest (RF), and XGBoost (Park et al. 2025). Furthermore, there is an urgent need to apply these advanced platforms to challenging therapeutic classes, such as anticancer drugs, where functional cardiotoxicity often occurs in the absence of overt structural damage or cell death. Importantly, many of these effects are time dependent, necessitating long-term monitoring (e.g., 120 h) to detect liabilities that are frequently missed by conventional short-term assays.

In this study, we developed and validated a robust cardiotoxicity screening platform that integrates high-purity ventricular-like hiPSC-CMs with an optimized AI learning model. We initially established the predictive performance of the CiPA 28 reference compounds. Our approach leverages AI to decode the complex crosstalk between multiple biomarkers, including the beat period, FPDcF, spike amplitude, and beating irregularity. By integrating diverse electrophysiological features, our model captures complex, non-linear patterns of cardiotoxicity, achieving an Receiver Operating Characteristic-Area Under Curve (ROC-AUC) of 0.982, which markedly exceeds that of existing single-biomarker or linear modeling approaches. Subsequently, we applied this validated model to evaluate the cardiotoxic profiles of various anticancer agents, including anthracyclines, tyrosine kinase inhibitors (TKIs), and chemotherapeutic agents. By differentiating between impedance-based cytotoxicity and MEA-based functional irregularities, we demonstrated that this integrated AI-hiPSC-CM platform can identify hidden proarrhythmic risks that conventional viability assays miss, providing a human-relevant tool to bridge the gap between preclinical screening and clinical cardiac safety.

## Materials & methods

### Human induced pluripotent stem cell-derived cardiomyocytes

Commercially available hiPSC-CMs were used in this study (Cardiosight^®^-S; NEXEL, South Korea). Cryopreserved Cardiosight^®^-S were thawed, plated, and maintained strictly per the manufacturer’s recommended protocols. Cell culture plates were coated with fibronectin (Sigma-Aldrich, F1141) diluted in phosphate-buffered saline to a final concentration of 50 µg/mL. The coated plates were incubated at 37 °C in a humidified atmosphere containing 5% CO_2_ for 1 h prior to cell seeding. After thawing, Cardiosight^®^-S were plated at the seeding density recommended by the manufacturer. Following cell attachment, the culture medium was replaced every 2 days. All medium changes were carefully performed to minimize mechanical stress on the cells. hiPSC-CMs were used for the experiments starting from day 7 after plating, allowing sufficient time for cellular recovery, attachment, and functional stabilization. Functional and electrophysiological analyses, including drug treatment, were initiated only after the stabilization period.

### MEA recordings of CiPA reference compounds

Electrophysiological responses to the CiPA reference compounds were evaluated using an MEA system (Axion Biosystems). hiPSC-CMs were plated onto MEA plates at a density of 5 × 10^4^ cells/well and maintained for 7 days prior to the experiments. MEA recordings were performed under physiological conditions (37 °C, 5% CO_2_). Before drug treatment, the cells were equilibrated in the MEA system for 30 min, and baseline recordings were obtained in the absence of the compounds. Twenty-eight CiPA reference compounds were sequentially tested at concentrations corresponding to 0.1, 1, 10, and 100× of their respective clinical maximum plasma concentrations (C_max_). Dimethyl sulfoxide (DMSO) was used as a negative control, and the final DMSO concentration was kept constant across all experimental conditions. For each concentration, one-tenth of the culture medium volume was removed and replaced with an equal volume of medium containing the compound at the appropriate concentration. Following the addition of each drug addition, the cells were allowed to stabilize for 30 min before MEA recording. MEA data were acquired to assess the beat period, FPDcF, spike amplitude, and beating irregularity, and were expressed as the coefficient of variation (CoV). The recorded signals were extracted and analyzed using AxIS Navigator software (Axion Biosystems). Detailed information on the CiPA 28 reference drugs and anticancer drugs used in this study is summarized in Tables 1 and 2.

**Table 1:** CiPA 28 reference drugs used in this study

CiPA TdP category	Drugs	Cmax (μM)	Final concentrations (μM)
High	Azimilide	0.07	0.007, 0.07, 0.7, 7
	Bepridil	0.03	0.003, 0.03, 0.3, 0
	Sotalol	15	1.5, 15, 150, 1500
	Disopyramide	0.7	0.07, 0.7, 7, 70
	Dofetilide	0.002	0.0002, 0.002, 0.02, 0.2
	Ibutilide	0.1	0.01, 0.1, 1, 10
	Quinidine	3	0.3, 3, 30, 300
	Vandetanib	0.3	0.03, 0.3, 3, 30
Intermediate	Astemizole	0.00003	0.000003, 0.00003, 0.0003, 0.003
	Chlorpromazine	0.035	0.0035, 0.035, 0.35, 3.5
	Cisapride	0.003	0.0003, 0.003, 0.03, 0.3
	Clarithromycin	1.2	0.12, 1.2, 12, 120
	Clozapine	0.07	0.007, 0.07, 0.7, 7
	Domperidone	0.02	0.002, 0.02, 0.2, 2
	Droperidol	0.02	0.002, 0.02, 0.2, 2
	Ondansetron	0.37	0.037, 0.37, 3.7, 37
	Pimozide	0.0004	0.00004, 0.0004, 0.004, 0.04
	Risperidone	0.002	0.0002, 0.002, 0.02, 0.2
	Terfenadine	0.0003	0.00003, 0.0003, 0.003, 0.03
Low/no risk	Diltiazem	0.13	0.013, 0.13, 1.3, 13
	Loratadine	0.0005	0.00005, 0.0005, 0.005, 0.05
	Metoprolol	1.8	0.18, 1.8, 18, 180
	Mexiletine	2.5	0.25, 2.5, 25, 250
	Nifedipine	0.008	0.0008, 0.008, 0.08, 0.8
	Nitrendipine	0.003	0.0003, 0.003, 0.03, 0.3
	Ranolazine	1.9	0.19, 1.9, 19, 190
	Tamoxifen	0.02	0.002, 0.02, 0.2, 2
	Verapamil	0.05	0.005, 0.05, 0.5, 5

**Table 2:** Anticancer drugs used in this study

No	Drugs	Category	Target	Cardiotoxicity side effect	Final concentrations (µM)
1	Doxorubicin	Anthracyclines	Breast cancer, bladder cancer, lymphocytic leukemia, used together with other chemotherapy agents	Cardiomyopathy and heart failure von Hoff et al. (1979), Feijen et al. (2015), Chan et al. (2023), Qianqian et al. (2024)	0.3, 1, 3
2	Daunorubicin	Anthracyclines	AML, ALL, CML and Kaposi’s sarcoma	Cardiomyopathy and heart failure Feijen et al. (2015), Carrasco et al. (2021), Chan et al. (2023)	0.3, 1, 3
3	Epirubicin	Anthracyclines	Breast cancer	Cardiomyopathy and heart failure Ryberg et al. (1998), Chan et al. (2023), Qianqian et al. (2024)	3, 10, 30
4	Idarubicin	Anthracyclines	Leukemia	Heart failure, heart attack, and abnormal heartbeats Anderlini et al. (1995), Qianqian et al. (2024)	0.00003, 0.0001, 0.0003
5	Sunitinib	Tyrosine kinase inhibitor	Renal cell carcinoma, GIST	Mitochondrial damage, cardiomyocyte apoptosis, hypertension Chu et al. (2007), Kerkela et al. (2009), Chan et al. (2023)	0.1, 0.3, 1
6	Sorafenib	Tyrosine kinase inhibitor	Lung cancer, kidney cancer, Liver cancer, AML, RAIR	> 10% frequency (hypophosphataemia, haemorrhage) 1 –10% (congestive heart failure, myocardial infarction, myocardial ischaemia) 0.01 – 0.1% (QT interval prolongation) Chan et al. (2023), Li et al. (2024)	3, 10, 30
7	Erlotinib	Tyrosine kinase inhibitor	NSCLC, Pancreatic cancer	Heart failure, heart attack, atrial fibrillation, and arrhythmia Waliany et al. (2021), Chan et al. (2023)	1, 3, 10
8	Vandetanib	Tyrosine kinase inhibitor	thyroid cancer	Irregular heartbeat and heart failure Zang et al. (2012), Scheffel et al. (2013)	1, 3, 10
9	Crizotinib	Tyrosine kinase inhibitor	NSCLC, ALCL, IMT	Irregular heartbeat Waliany et al. (2021); Chan et al. (2023)	0.3, 1, 3
10	Cisplatin	Chemotherapy	Testicular cancer, ovarian cancer, cervical cancer, bladder cancer etc	Heart damage Hu et al. (2018), Chan et al. (2023)	10, 30, 100
11	Carboplatin	Chemotherapy	ovarian cancer, lung cancer, head and neck cancer, brain cancer	Heart damage Chan et al. (2023)	30, 100, 300
12	Cyclophosphamide	Chemotherapy	lymphoma, multiple myeloma, leukemia, ovarian cancer, breast cancer	Cardiotoxicity with higher dose regimens Ryberg et al. (1998), Chan et al. (2023)	3, 10, 30

### Data analysis and statistics

Quantitative analysis of the fluorescence images was performed using ImageJ software. FPDcF was calculated using Fridericia’s formula (von Hoff et al. 1979). Drug-induced responses over time were expressed as percentage changes relative to baseline values. All measurements were performed with at least three technical replicates per condition. Statistical analyses were performed using the GraphPad Prism software (version 10.6.1). Comparisons between groups were conducted using one-way or two-way analysis of variance, as appropriate, followed by post hoc multiple comparison tests. Values with *P* < 0.05 were considered statistically significant.

## Results

### Phenotypic characterization of hiPSC-CMs

**Fig. 1:**
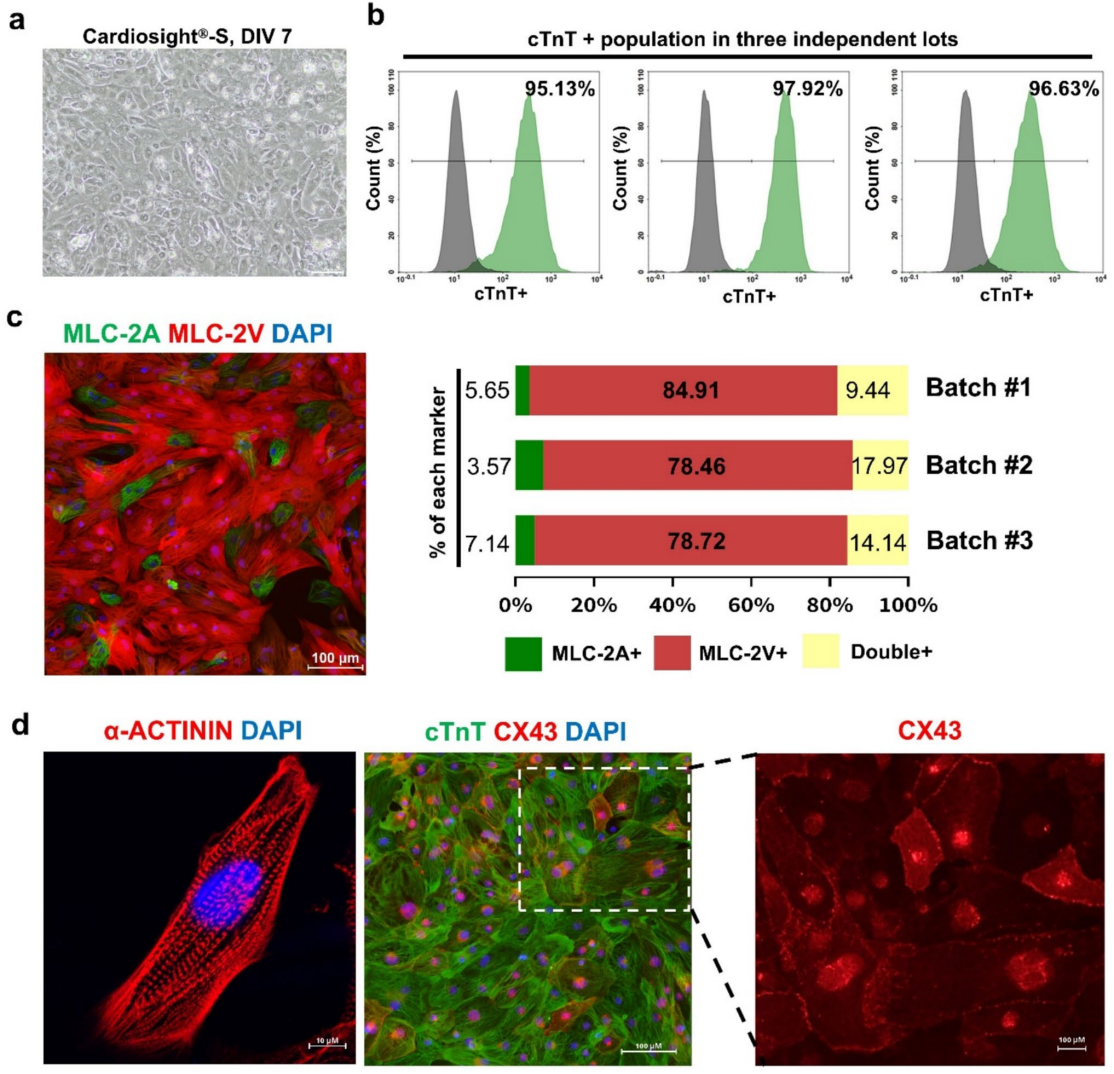
Phenotypic and functional characterization of human induced pluripotent stem cell-derived cardiomyocytes (hiPSC-CMs). **a** Representative morphology of hiPSC-derived cardiomyocytes. Bright-field microscopy image of hiPSC-CMs (Cardiosight^®^-S) at day in vitro (DIV) 7 post-thawing. **b** Flow cytometric assessment of cardiac purity. Representative histograms and quantitative analysis of cardiac troponin T (cTnT +) expression across three independent batches. **c** Subtype-specific characterization and quantification. Representative immunofluorescence images for ventricular (MLC-2 V, red) and atrial (MLC-2A, green) markers at DIV 7. The stacked bar graph demonstrates a predominant ventricular-like population, with MLC-2 V + cells across three independent batches. (**d**) Structural maturity and functional coupling markers. High-magnification immunofluorescence staining for sarcomeric α-actinin (red), cardiac troponin T (cTnT, green), and the gap junction protein Connexin 43 (CX43, red)

To establish biological relevance and ensure batch-to-batch consistency for high-fidelity safety assessments, we conducted a rigorous phenotypic characterization of cryopreserved hiPSC-CMs (Cardiosight^®^-S). Upon thawing and subsequent culturing, hiPSC-CMs reached full confluence by days in vitro (DIV) 7, exhibiting robust, well-synchronized spontaneous contractions (Fig. 1a). Flow cytometric analysis of the pan-cardiac marker cardiac troponin T (cTnT) confirmed exceptionally high and reproducible differentiation efficiency across three independent batches: 95.13% (batch #1), 97.92% (batch #2), and 96.63% (batch #3) (Fig. 1b). Furthermore, subtype-specific analysis revealed a predominant ventricular-like identity, with MLC-2 V + populations ranging from 78.46 to 84.91% (Fig. 1c). Immunostaining further validated the structural maturity and integrity of the cells, demonstrating well-organized sarcomeric patterns via α-actinin and the distinct expression of the gap junction protein Connexin 43 (Fig. 1d). These results demonstrated the development of a functionally connected and highly integrated myocardial syncytium, offering a dependable, human-relevant basis for electrophysiological drug screening.

### Validation of AI-based proarrhythmic risk prediction

**Fig. 2:**
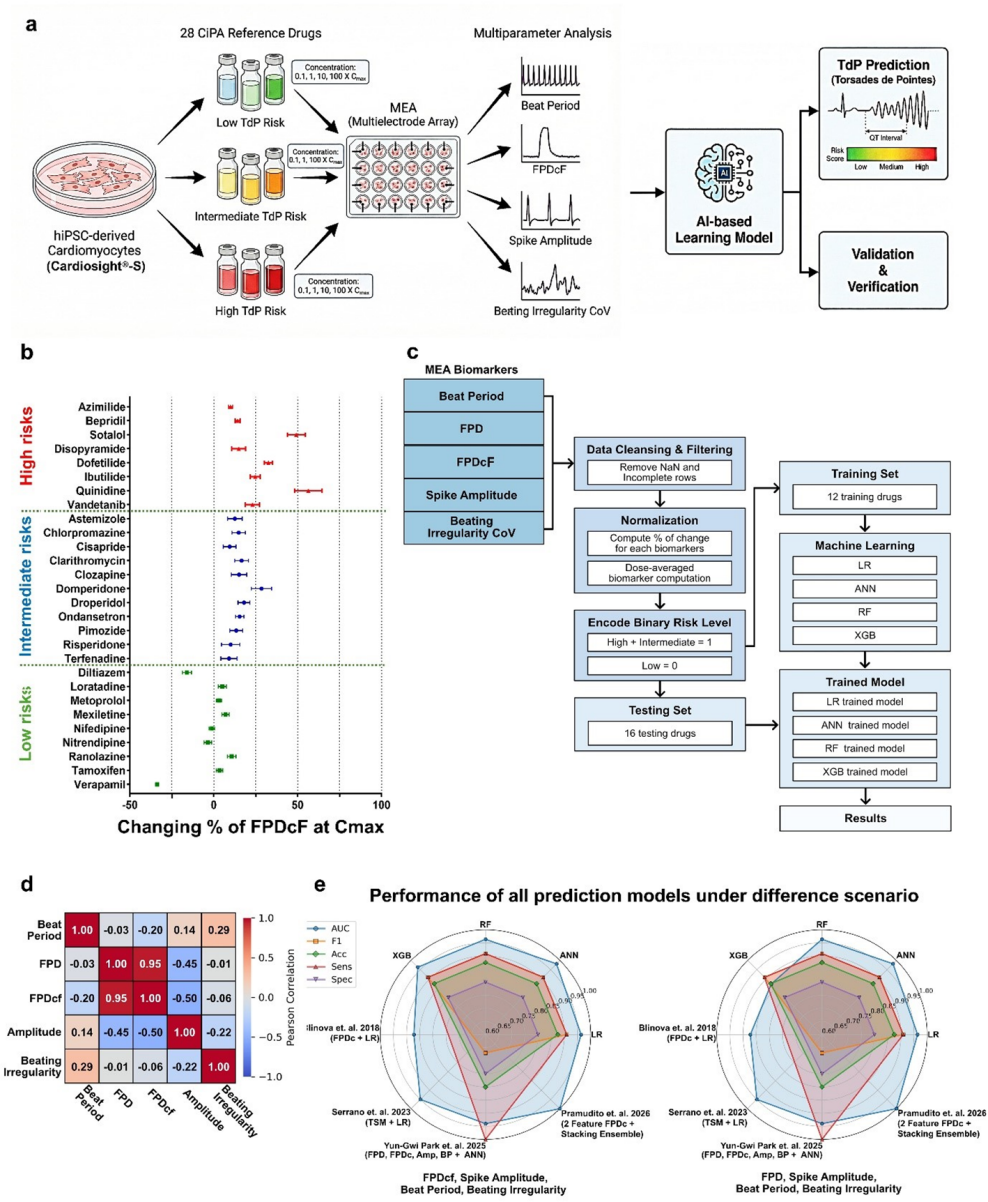
AI-driven validation of proarrhythmic risk assessment using the CiPA 28 Reference Compounds in hiPSC-CMs. **a** Integrated workflow for cardiac safety evaluation. Schematic representation of the platform combining hiPSC-CMs (Cardiosight^®^-S) with an AI-based learning model. This involves treating cells with 28 CiPA reference compounds at multiple concentrations (0.1–00 X Cmax), followed by high-content Multielectrode Array (MEA) data acquisition and multi-parametric analysis to train and verify TdP prediction models. **b** Electrophysiological responses of CiPA reference compounds. Distribution of the percentage change in corrected field potential duration (FPDcF) at Cmax for the 28 reference drugs. The results show that FPDcF prolongation or shortening closely aligns with clinical TdP risk categories (High, Intermediate, and Low risk), confirming the electrophysiological sensitivity of the hiPSC-CM (Cardiosight^®^-S) platform. **c** Workflow schematic of the AI-integrated cardiotoxicity screening platform. MEA biomarkers are preprocessed through data cleaning, normalization, and ensemble binary classification, then divided into training (12 CiPA compounds) and testing sets (16 CiPA compounds). Four machine learning models (LR, ANN, RF, XGB) are trained on the training dataset and subsequently evaluated on the held-out test set. **d** Pearson correlation matrix of MEA biomarkers. The heatmap displays pairwise correlations among the four MEA-derived biomarkers (beat period, FPD, FPDcF, spike amplitude, and beating irregularity), with correlation coefficients ranging from 0 to 1 shown in each cell. FPD and FPDcF exhibit the highest correlation (r = 0.95), while correlations between FPDcf/FPD and the other biomarkers remain below 0.30, indicating complementary information captured by each parameter. **e** Performance comparison of four machine learning models under Scenario 1 (FPDcF-based) and Scenario 2 (FPD-based) biomarker combinations. Radar plots display median performance metrics (AUC, F1-score, accuracy, sensitivity, specificity) for LR, ANN, RF, and XGB models trained with either FPDcF or uncorrected FPD as the primary repolarization biomarker. All Scenario 1 models achieved AUC values exceeding 0.95, while Scenario 2 XGB performance fell below 0.90, demonstrating the superior performance of rate-corrected FPDcF over uncorrected FPD

To validate the utility of Cardiosight^®^-S for cardiac safety evaluation, we assessed the electrophysiological responses to the 28 CiPA reference compounds using the MEA platform (Fig. 2a) High-content biomarkers, including beat period, FPDcF, spike amplitude, and beating irregularity (CoV) were quantified before and after drug exposure to capture the complex ionic interactions induced by the compounds. Analysis of FPDcF changes at C_max_ concentrations demonstrated that the degree of prolongation or shortening was highly correlated with the established clinical TdP risk classifications (Fig. 2b). Specifically, high-risk drugs induced a significant extension of the repolarization phase, whereas low-risk compounds showed minimal deviations or anticipated shortening effects.

To classify the proarrhythmic risk, we developed AI-driven predictive models using four distinct machine learning architectures. These models were trained on comprehensive MEA datasets from 12 CiPA reference compounds and subsequently validated on 16 held-out test compounds (Fig. 2a, c, and Supplementary Fig. 1a). Correlation analysis of the inputs revealed a strong correlation between FPD and FPDcF (r = 0.95) (Fig. 2d). In contrast, the correlations between these parameters and other biomarkers remained modest (r < 0.30), suggesting that these features provided complementary information for risk prediction. To rigorously evaluate the model’s generalization, we implemented a repeated-sampling strategy with 10,000 independent test iterations. We compared two distinct multi-biomarker scenarios to determine the optimal combination for classifyng TdP risk. Scenario 1 used FPDcF, spike amplitude, beat period, and beating irregularity, whereas scenario 2 used uncorrected FPD instead. In Scenario 1 (FPDcF-based), all four models consistently exhibited a high performance, with a median AUC value exceeding 0.95. The ANN achieved a median AUC of 0.982, XGBoost achieved a median AUC of 0.964, and Logistic Regression (LR) achieved a median AUC of 0.964 (Fig. 2e, Supplementary Fig. 1b). In Scenario 2 (FPD-based), the RF achieved a median AUC of 0.964, ANN achieved a median AUC of 0.982, XGBoost achieved median AUC of 0.873, and LR achieved median AUC of 0.964 (Fig. 2e and Supplementary Fig. 1b). Detailed performance metrics across all 10,000 iterations, including median accuracy, sensitivity, specificity, F1-score, and their 95% confidence intervals, are presented in Supplementary Fig. 1b. Furthermore, the radar plots in Fig. 2e provide a comprehensive visualization of the model performance across the evaluation metrics for both scenarios. Collectively, these findings confirm that our integrated AI-hiPSC-CM platform is a highly specific and quantitative tool for predicting clinical proarrhythmic risk, offering significantly greater sensitivity than relying solely on a single FPDcF parameter.

### Cytotoxicity profiling of anticancer agents

**Fig. 3:**
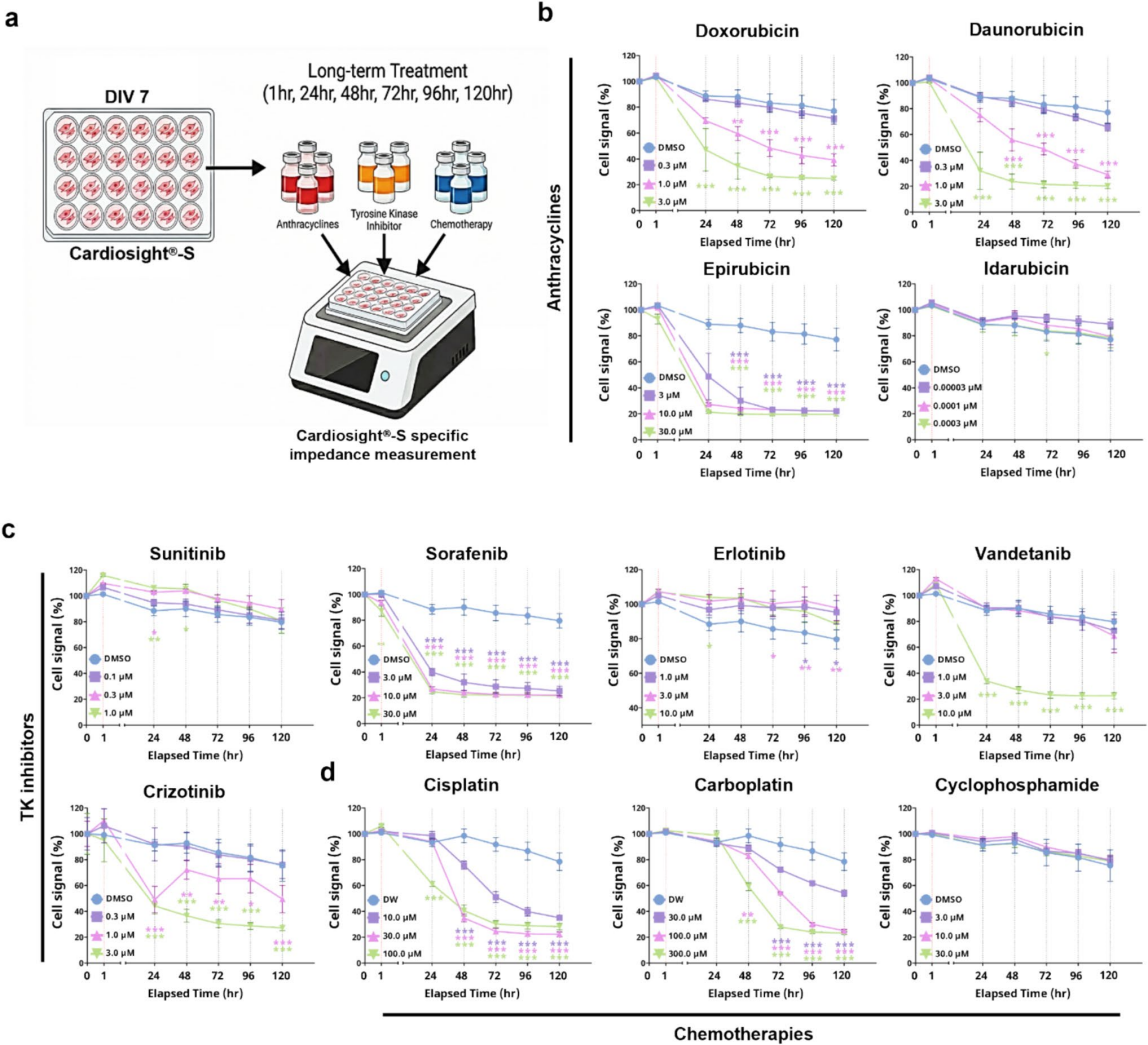
Cytotoxicity profiling of anticancer agents in hiPSC-CMs. **a** Experimental workflow for impedance-based cytotoxicity assessment. Schematic representation of the long-term (up to 120 h) structural cardiotoxicity screening using hiPSC-CMs (Cardiosight^®^-S) integrated with the AltaZ platform. The platform enables continuous, label-free monitoring of cardiomyocyte health and viability following exposure to various classes of anticancer drugs. **b** Impact of Anthracyclines on hiPSC-CM specific signals. Time-dependent changes in specific impedance signals (%) following treatment with anthracyclines, including Doxorubicin, Daunorubicin, Epirubicin, and Idarubicin. **c** Impact of Tyrosine Kinase (TK) inhibitors on hiPSC-CM specific signals. Real-time impedance monitoring of hiPSC-CMs treated with various TKIs (Sunitinib, Sorafenib, Erlotinib, Vandetanib, and Crizotinib. **d** Impact of Chemotherapies on hiPSC-CM specific signals. Impact of cisplatin, carboplatin, and cyclophosphamide on cardiomyocyte viability. Statistical significance among treatment groups was defined as *P* < 0.05 using one-way ANOVA

To evaluate the predictive utility of the hiPSC-CM platform for clinically relevant cardiac risks, 12 anticancer agents with documented cardiotoxicity or potential clinical safety concerns (Fig. 3a). This panel included four anthracyclines (doxorubicin, daunorubicin, epirubicin, and idarubicin) (Fig. 3b), five TKIs (sunitinib, sorafenib, erlotinib, vandetanib, and crizotinib) (Fig. 3c), and three conventional chemotherapies (cisplatin, carboplatin, and cyclophosphamide) (Fig. 3d). To assess structural cardiotoxicity, we measured changes in Cardiosight^®^-S-specific impedance signals over a long-term exposure period of up to 120 h using Nanion AtlaZ platform. Building upon a preliminary optimization study, impedance measurements commenced on day 7 post-plating to ensure a Cardiosight^®^-S specific frequency (Supplementary Fig. 2). A sampling frequency of 9.6 kHz was identified as optimal for subsequent analyses, as it exhibited minimal inter-batch variability and provided the maximum dynamic range (gap value) throughout the culture period up to day 14. This optimized approach allowed for the sensitive detection of subtle changes in hiPSC-CM-based impedance, reflecting the early stages of drug-induced structural remodeling in human cardiomyocytes.

Continuous impedance-based monitoring showed that most agents caused significant reductions in cell signals over dose and time, indicating clear structural damage (Fig. 3b and c). However, four specific compounds (idarubicin, erlotinib, sunitinib, and cyclophosphamide) exhibited minimal to no significant reduction in impedance signals at the tested concentrations throughout the 120-h duration (Fig. 3b, c and d). These findings highlight a critical limitation of relying solely on viability-based screening. While these agents are clinically associated with cardiac adverse events, their cardiotoxicity may manifest as functional impairments rather than immediate structural cell death. These results underscore the necessity for integrated functional assessments to effectively identify the full spectrum of drug-induced cardiotoxic risks.

### Identification of hidden functional cardiotoxicity of anticancer agents

**Fig. 4:**
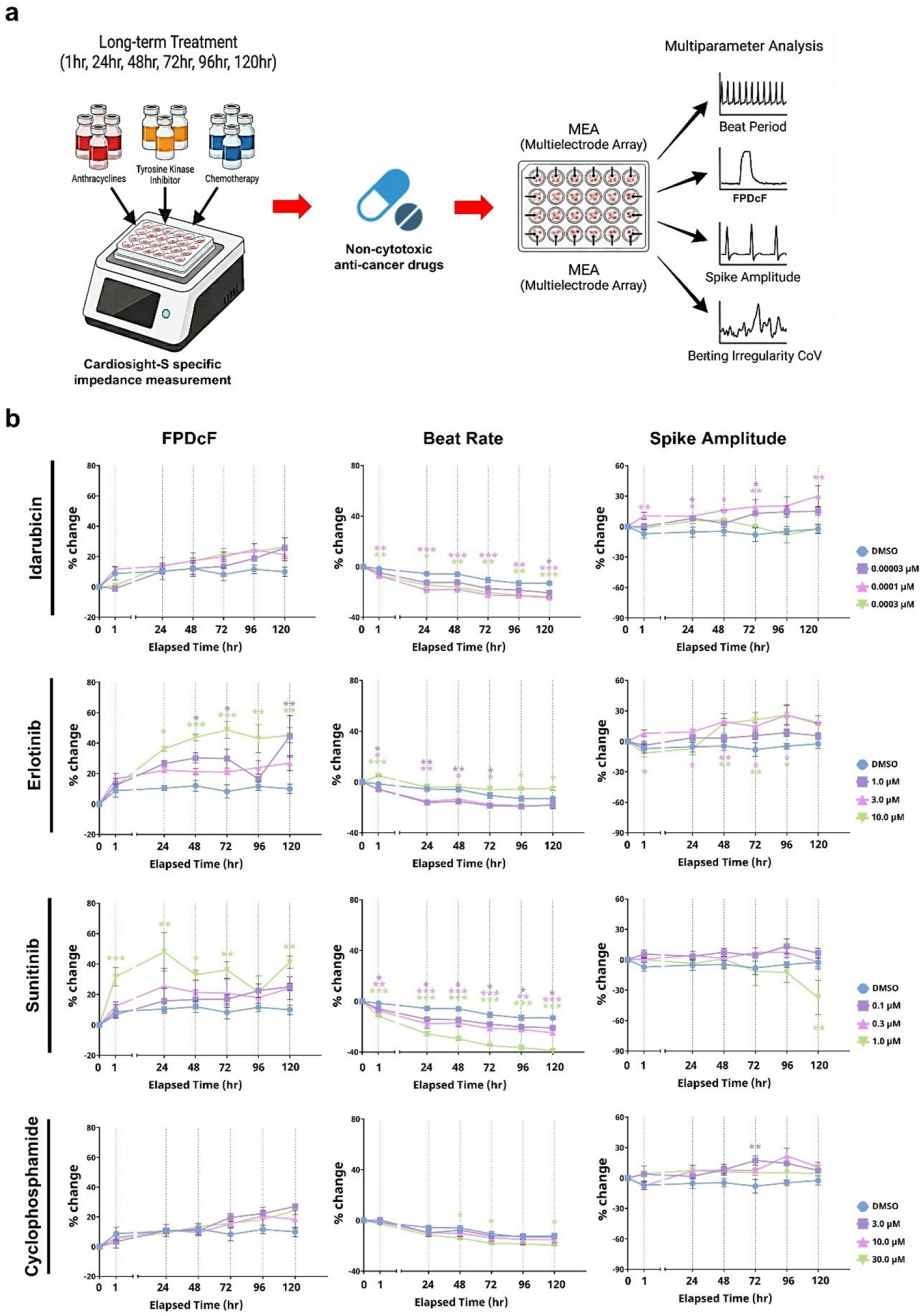
Electrophysiological Impact of Non-cytotoxic Anticancer Drugs on FPDcF, Beat Rate, and Spike Amplitude. **a** Experimental workflow for functional assessment. Schematic representation of the transition from impedance-based cytotoxicity screening to detailed electrophysiological profiling using the Multielectrode Array (MEA) platform. **b** Time-dependent electrophysiological alterations. Longitudinal analysis of corrected field potential duration (FPDcF), beat rate, and spike amplitude in hiPSC-CMs (Cardiosight^®^-S) following exposure to the selected anticancer agents. Data is presented as the percentage change (%) relative to DMSO-treated controls over a 120-h period. Statistical significance among treatment groups was defined as *P* < 0.05 using one-way ANOVA

Following the identification of the compounds that failed to exhibit observable structural cytotoxicity, we performed high-fidelity electrophysiological profiling of Idarubicin, Erlotinib, Sunitinib, and Cyclophosphamide. To capture subtle functional perturbations, MEA was performed with the same concentration ranges and temporal parameters as in the previous impedance-based cytotoxicity assays (Fig. 4a).

While most of the tested agents maintained stable contractile parameters (beat rate and spike amplitude) during long-term treatment, sunitinib emerged as a distinct exception. At its maximum concentration (1 µM), sunitinib induced an approximately 40% reduction in both beat rate and spike amplitude following 120 h of exposure (Fig. 4b). This pronounced decline suggests a significant impact on the myocardial contractile kinetics that occurs independently of cell death.

Significant alterations in the FPDcF were observed for all four compounds. Both idarubicin and cyclophosphamide exhibited marked FPDcF prolongation compared to the DMSO controls starting at 72 h of treatment (Fig. 4b). Specifically, idarubicin (0.0001 and 0.0003 µM) resulted in prolongation exceeding 20% after 96 h, while cyclophosphamide showed a consistent > 20% increase across all concentrations starting from the 72-h, indicating a high potential for proarrhythmic risk. Erlotinib and sunitinib induced the most rapid and significant delays in repolarization. Erlotinib induced an FPDcF increase exceeding 20% across all treatment groups compared to controls, while sunitinib (0.3 and 1 µM) exceeded this 20% threshold within only 24 h of treatment (Fig. 4b).

**Fig. 5:**
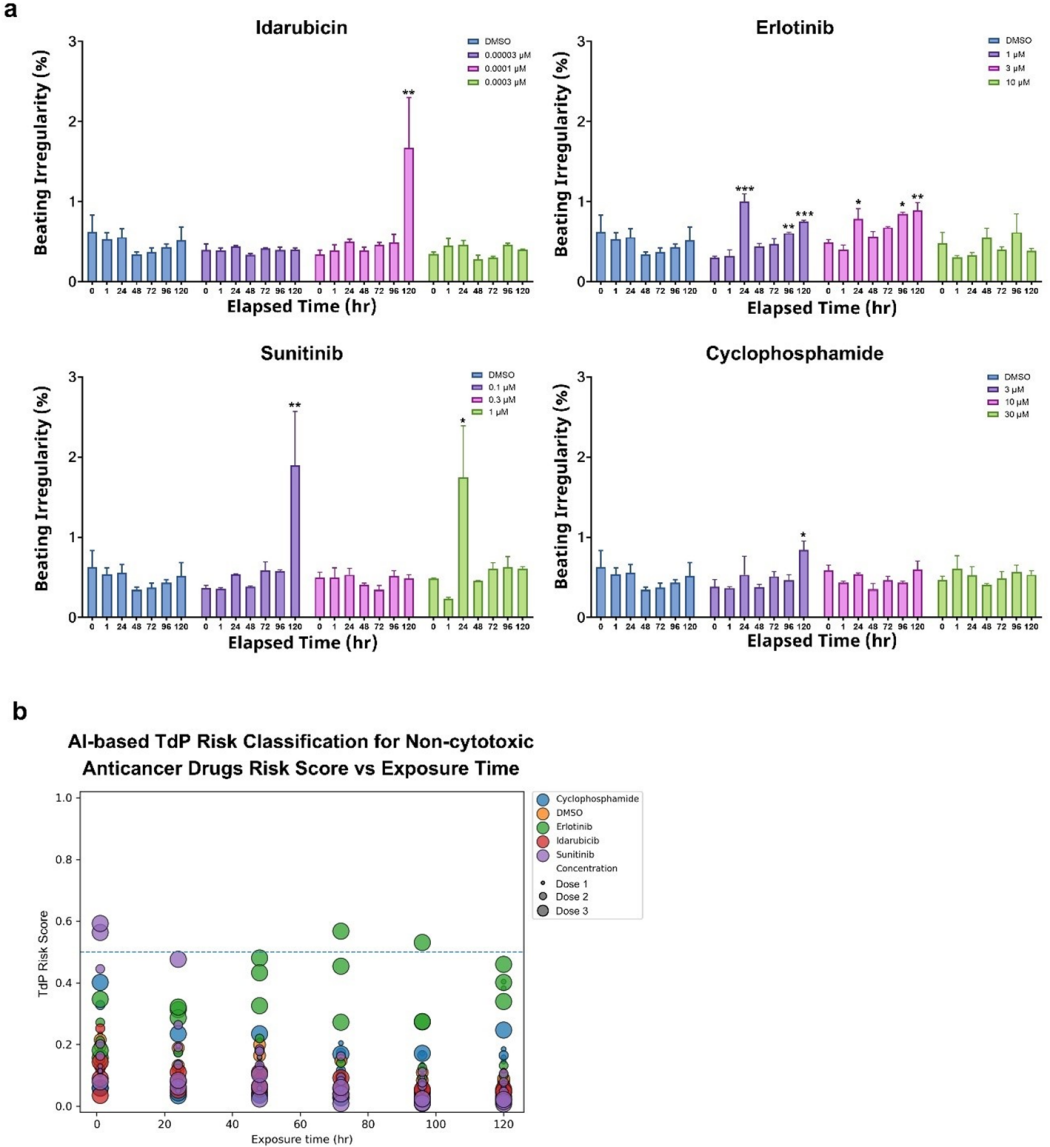
Beating irregularity analysis and AI-based TdP risk classification for non-cytotoxic anticancer drugs. **a** Beating irregularity analysis in MEA assay after treatment of selected anticancer agents on hiPSC-CMs (Cardiosight^®^-S). MEA-based assessment of beating irregularity (%) in hiPSC-CMs (Cardiosight^®^-S) following long-term exposure to sub-cytotoxic concentrations of the selected anticancer agents. Statistical significance among treatment groups was assessed using one-way ANOVA with *P* < 0.05 as the criterion. **b** TdP risk stratification of non-cytotoxic anticancer drugs through soft-voting ensemble classifier. Ensemble risk scores (ranging from 0 to 1.0) are plotted against exposure time (1–120 h) across multiple concentrations for four anticancer agents

Assessment of beating irregularities (CoVs) yielded critical evidence of functional instability. Notable increases in irregularity were detected following 120 h of treatment with 0.0001 µM idarubicin and 0.1 µM sunitinib (Fig. 5a). Furthermore, a significant increase in irregularity was observed as early as 24 h after treatment with 1 µM sunitinib (Fig. 5a).

Following validation of the CiPA reference compounds, we applied the validated Scenario 1 soft-voting ensemble classifier to evaluate the proarrhythmic risk of the four non-cytotoxic anticancer agents (Fig. 5b). Our analysis revealed that significant FPDcF prolongation in the MEA assays was classified as indicating a high to intermediate risk for TdP. Sunitinib was classified as high/intermediate risk for TdP, with ensemble risk scores > 0.5 at early time points and high concentrations. Erlotinib was similarly classified as high/intermediate risk, demonstrating ensemble risk scores > 0.5 at later time points (≥ 72 h) and across higher concentrations, with risk scores escalating as exposure duration increased. In contrast, idarubicin and cyclophosphamide were both classified as low risk, with ensemble risk scores remaining below 0.5 throughout the 120-h exposure period and across all tested concentrations. The temporal dynamics of the ensemble risk scores indicated an increase in proarrhythmic risk during the long-term exposure assessment, whereas idarubicin and cyclophosphamide consistently remained at low risk, regardless of exposure duration and concentration. Collectively, these findings demonstrate that our integrated AI-hiPSC-CM system is a more sensitive tool for TdP risk assessment than conventional single-parameter FPDcF analysis. By integrating multiple electrophysiological biomarkers, this platform enabled the detection of time-dependent cardiotoxic risks that require long-term monitoring to ensure patient safety during pharmacotherapy.

## Discussion

The primary challenge in preclinical drug safety is accurately predicting human-specific cardiotoxicity, an area where traditional animal models and hERG assays frequently fall short. Our study addresses this gap by integrating high-purity, ventricular-like hiPSC-CMs with advanced AI modeling, providing a robust platform that aligns with the CiPA and ICH E14/S7B regulatory frameworks. A critical strength of our hiPSC-CM model was its high ventricular-like purity, with approximately 80% of MLC-2 V + cells observed across multiple batches. Given that TdP is fundamentally a ventricular phenomenon (Anderlini et al. 1995; Chu et al. 2007), the use of such subtype-specific cells significantly enhances the physiological relevance of the recorded MEA signals.

A key highlight of our study is the better predictive performance achieved through our AI-integrated approach compared to existing research. In a landmark multi-site study, Blinova et al. (2018) utilized FPDcF as a single biomarker for TdP risk classification, reporting a ROC-AUC of approximately 0.89. Furthermore, more recent advancements by Serrano et al. (2023) demonstrated that machine learning models could enhance prediction accuracy by incorporating multiple MEA features. In contrast to conventional methods, our platform distinguishes itself by leveraging a comprehensive, multi-parametric dataset that incorporates the beat period, FPD, FPDcF, spike amplitude, and beating irregularity. By leveraging these diverse electrophysiological features, the system achieved a significantly higher ROC-AUC > 0.95 than that of the CiPA 28 reference drugs. This performance markedly exceeds that of the single-biomarker approach established by Blinova et al. (2018) and various machine learning architectures recently explored by Serrano et al. These findings underscore the fact that the synergy between high-purity biological substrates (hiPSC-CMs) and sophisticated AI integration enables a significantly more nuanced and accurate interpretation of cardiac safety than previous methods. By integrating diverse electrophysiological features, the model captures complex, non-linear patterns of cardiotoxicity, which are often obscured when relying on single-parameter thresholds.

Our findings on anticancer drugs provide further insights into the divergence between functional and structural cardiotoxicities. Although many TKIs, anthracyclines, and chemotherapies are known to induce chronic heart failure via structural damage (Kerkela et al. 2009), our data revealed that anticancer agents, such as idarubicin, erlotinib, sunitinib, and cyclophosphamide can trigger potent proarrhythmic signals. These functional disturbances are marked by changes in the FPDcF, beat rate, spike amplitude, and irregularity of beating. Importantly, these electrophysiological alterations appear even at subcytotoxic levels that do not cause immediate cell death, emphasizing a critical window of hidden cardiotoxicity that conventional viability-based screening might miss.

Building upon these multi-parametric observations of non-cytotoxic anticancer drugs, our AI-driven predictive model translates these complex, non-linear electrophysiological signals from MEA analysis into a standardized TdP risk score, enabling a level of precision that exceeds traditional binary classifications. By calculating temporal risk scores for sunitinib and erlotinib, the model effectively quantified their proarrhythmic liabilities and classified them as high-to-intermediate risk based on early-onset and time-dependent functional instabilities. Conversely, the consistently low risk scores assigned to idarubicin and cyclophosphamide demonstrated the platform’s high specificity in distinguishing between physiological fluctuations and genuine proarrhythmic threats. These findings demonstrate that integrating sophisticated machine learning with high-fidelity hiPSC-CMs enables the detection of subtle, time-dependent cardiotoxic signals that are frequently missed by conventional safety pharmacology assays.

**Fig. 6:**
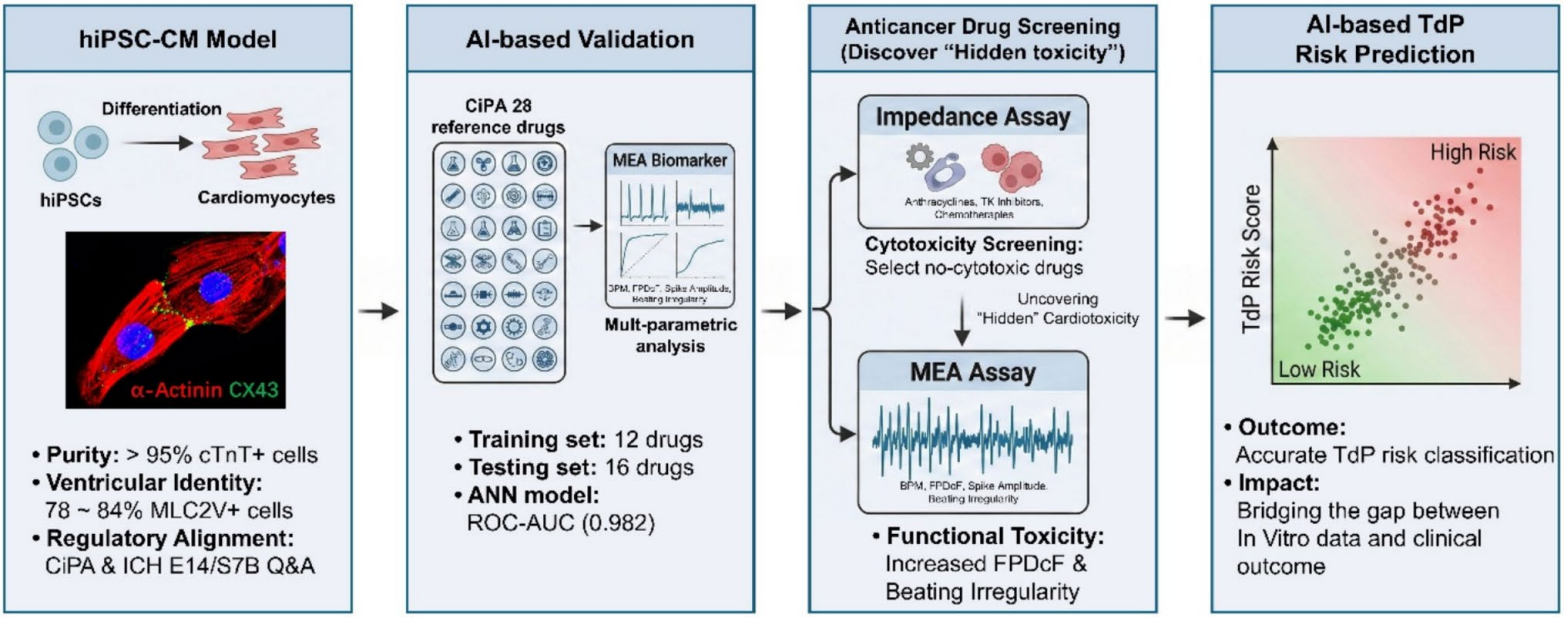
Integrated platform for enhanced cardiotoxicity screening using ventricular-like hiPSC-CMs and AI-driven predictive modeling

In conclusion, we developed and validated an integrated platform that combined high-purity, ventricular-like hiPSC-CMs with advanced AI-driven predictive modeling for enhanced cardiotoxicity screening (Fig. 6). Our results demonstrate that the biological fidelity of cardiomyocytes provides a reliable human-relevant substrate for electrophysiological assessments. By leveraging AI trained on CiPA 28 reference compounds, we achieved exceptional predictive accuracy and significantly improved TdP risk classification over traditional single-biomarker approaches.

## Conclusions

Our integrated AI-hiPSC-CM platform represents a significant advancement in the NAMs for cardiac safety assessments. By overcoming the limitations of single-parameter measurements and incorporating the temporal dynamics of ventricular-like hiPSC-CM behavior through sophisticated machine learning, we developed a more holistic and human-relevant screening strategy. This approach not only aligns with the ongoing global shift toward animal-free testing, as championed by the FDA Modernization Act 2.0, but also offers a proactive solution for identifying potential proarrhythmic liabilities earlier in drug development.

The ability to detect hidden cardiotoxicity in drugs like Sunitinib, Erlotinib, Idarubicin, and cyclophosphamide, which seem safe in standard tests, highlights the need to combine functional electrophysiological monitoring with predictive AI. Ultimately, the synergy demonstrated in this study paves the way for a more reliable, precise, and clinically predictive framework of pharmacological safety. By providing quantitative, standardized TdP risk scores, this platform ensures that potentially life-saving therapeutics can be developed with a comprehensive understanding of their cardiac safety profile, bridging the translational gap and enhancing patient safety in the clinic.

## Supplementary Information

Below is the link to the electronic supplementary material.

Supplementary file1 (PDF 694 KB)

## Data Availability

The datasets generated and/or analyzed in the current study are available from the corresponding author upon reasonable request.
