# Supplementary material for: Integrating high-fidelity hiPSC-cardiomyocytes with AI-driven modeling for enhanced proarrhythmic risk assessment
Source: Arch Toxicol. 2026 Apr 28;100(8):3553–65. doi: 10.1007/s00204-026-04361-8 (PMC13379419; doi:10.1007/s00204-026-04361-8)
Supplement: Supplementary file 1 — Supplementary file1 (PDF 694 KB) [file 204_2026_4361_MOESM1_ESM.pdf]

Integrating High-Fidelity hiPSC-Cardiomyocytes with AI-Driven Modeling for Enhanced Proarrhythmic Risk Assessment

Su-Bin Kim[1*], Jaehun Lee[1*], Jieun An[1], Ara Cho[1], Kun Hee Lee[1], Hwan Choi[1], Choongseong Han[1], Muhammad Adnan Pramudito[3], Ki Moo Lim[2,4#], and Dong-Hun Woo[1#]

[1]Department of Commercializing iPSC Technology, NEXEL Co., Ltd., Seoul, 07802, Republic of Korea.

[2]Department of Biomedical Engineering, Kumoh National Institute of Technology, Gumi, 39177, Republic of Korea.

[3]Department of IT Convergence Engineering, Kumoh National Institute of Technology, Gumi, 39177, Republic of Korea.

[4]Meta Heart Co., Ltd., Gumi, 39177, Republic of Korea.

*Contributed equally.

[#]Correspondence to:

Dong-Hun Woo, Ph.D.

[1]Department of Commercializing iPSC Technology, NEXEL Co., Ltd.,

8th floor, 55 Magokdong-ro, Gangseo-gu, Seoul 07802, Republic of Korea

dhwoo@nexel.co,kr

Ki Moo Lim., Ph.D.

[2]Department of Biomedical Engineering, Kumoh National Institute of Technology, Gumi, 39177, Republic of Korea.

[4]Meta Heart Co., Ltd., Gumi, 39177, Republic of Korea.

kmlim@kumoh.ac.kr.

Supplementary method

Morphological characterizations by microscopy

Morphological characterization of hiPSC-CMs was performed using a phase-contrast microscope (Nikon, TS100). Cells were observed starting from day 1 in vitro (DIV1), one day after thawing, and subsequently monitored at 2-day intervals. During each observation, cell density, the presence of non-cardiomyocyte populations, cellular aggregation, and the occurrence of spontaneous and synchronous contractile activity were qualitatively assessed and documented. Representative images were acquired on day 7 after plating using a 10× objective lens. Imaging locations were selected based on average cell density and beating activity across the well.

Flow cytometry analysis

Cardiomyocyte purity was evaluated by flow cytometry using cardiac troponin T (cTnT) staining. hiPSC-derived cardiomyocytes at day 7 in vitro (DIV7) were dissociated with TrypLE Express (Gibco, 12604021), neutralized with high glucose DMEM supplemented with 10 % FBS, and collected by centrifugation. Cells (4×10^5 per sample) were fixed using a commercial fixation solution (BD Biosciences, 554722) and permeabilized with 1× Wash/Perm solution (BD Biosciences, 554723). Intracellular staining was performed using an anti-cardiac troponin T antibody (Abcam, ab8295) or an isotype control, followed by incubation with an Alexa Fluor 488–conjugated secondary antibody (Invitrogen, A21202). Samples were analyzed using a NovoCyte® flow cytometer (Agilent Technologies) applying standard FSC/SSC and singlet gating strategies, and the percentage of cTnT-positive cells was quantified.

Immunofluorescence staining and microscopy

Immunofluorescence staining was performed using antibodies against MLC-2A (Synaptic Systems, 311011), MLC-2V (Abcam, ab799355), α-actinin (Abcam, ab9465), cardiac troponin T (cTnT; Abcam, ab8295), and connexin 43 (CX43; Abcam, ab11370). hiPSC-CMs were fixed with 4 % paraformaldehyde, permeabilized with 0.1 % Triton X-100, and blocked with 10 % donkey serum in DPBS. Primary antibodies were applied overnight at 4 °C, followed by incubation with appropriate fluorophore-conjugated secondary antibodies. Nuclei were counterstained using mounting medium containing DAPI (Vector Laboratories, H-1200-10). Fluorescence images were acquired using an Eclipse Ti2 inverted fluorescence microscope (Nikon) or a confocal laser scanning microscope (Carl Zeiss, LSM 900).

Cardiotoxicity assessment and long-term drug exposure

hiPSC-CMs were plated onto fibronectin-coated NSP-96 Type Standard Stim Plates (Nanion, #20-1003) and maintained under standard culture conditions. Culture medium was replaced every 2 days using a half-medium change protocol. During drug treatment, half of the medium volume was replaced with fresh medium containing compounds at the target concentrations to maintain consistent drug exposure. Impedance measurements were continuously recorded using the AtlaZ system (Nanion). Based on preliminary validation studies, hiPSC-CMs exhibited stable impedance signals for up to 14 days when measured at a frequency of 9.60 kHz. Therefore, impedance-based cardiotoxicity assessment was performed over a 120-h exposure period. Impedance values were normalized to baseline levels prior to drug exposures. Four anticancer drugs (idarubicin, erlotinib, sunitinib, and cyclophosphamide) were tested at five different concentrations, and impedance signals were collected continuously during drug treatment. As a negative control, cells were treated with DMSO alone at the same final concentration (0.1%) used in drug-treated conditions. To enable direct comparison between impedance-based and electrophysiological readouts, parallel cultures were maintained under MEA conditions and monitored for 120 h. MEA recordings were acquired at predefined time points (1, 24, 48, 72, 96, and 120 h) following drug exposure. The detailed information of anticancer drugs used in this study is summarized in table 2.

Data reprocessing and feature selection for AI machine learning

To prepare the comprehensive MEA-derived biomarker dataset for machine learning analysis, we implemented a systematic preprocessing pipeline that prioritized both data integrity and biological relevance (Fig. 2c). Initial data cleaning involved removal of samples with missing values (NaN) or incomplete biomarker recordings across any of the five biomarkers examined including FPDcF, field potential duration (FPD), beat period, spike amplitude, and beating irregularity (coefficient of variation, CoV). This conservative approach prioritized data quality over quantity, as missing values may indicate technical failures during MEA acquisition that would compromise electrophysiological interpretation and confound subsequent machine learning predictions.

All biomarker measurements were then normalized by calculating percentage change relative to vehicle control (DMSO-treated cells) using the following Eq. 1 (Serrano et al. 2023).

$$\%\Delta Biomarkers = \frac{Biomarkers_{drug} - Biomarkers_{baseline}}{Biomarkers_{baseline}} \times 100 \qquad \text{Eq. 1}$$

This normalization strategy is standard in electrophysiological pharmacology and reduces technical variability inherent in MEA recordings, including measurement noise, electrode drift, and batch-to-batch effects, while preserving biologically relevant drug-induced signals. Given the limited sample size per condition consisting of 4 technical replicates and 4 concentrations per drug, equaling 16 raw data points per drug, we implemented a data augmentation strategy designed to generate meaningful additional training instances without compromising data integrity. Specifically, for each drug, we created multiple combinations by systematically averaging normalized biomarker values across different sample-concentration windows, thereby modeling the integrated pharmacological response across the entire concentration range tested (Park et al. 2025). This approach is mechanistically grounded in pharmacological principles, as a drug's clinical risk profile reflects its integrated effect across therapeutically relevant concentrations rather than effects at isolated doses (Park et al. 2025). The averaging procedure thereby models dose-accumulation and temporal integration of cardiac effects, two key determinants of whether proarrhythmic risk emerges. This augmentation strategy offered multiple advantages in that it increased training data volume without artificial duplication, it preserved the biological meaning of each observation by remaining within the measured concentration range, and it explicitly modeled dose-dependency inherent to pharmacological responses. This methodology aligns with established practice in drug discovery, where limited datasets require intelligent augmentation to enhance model generalization while maintaining mechanistic plausibility.

Following data augmentation, we performed class label encoding by assigning compounds classified as high-risk or intermediate-risk to category 1 and low-risk compounds to category 0, a binary encoding strategy that aligns with clinical decision-making frameworks distinguishing drugs requiring cardiac safety mitigation from those with acceptable proarrhythmic profiles. Prior to model training, Pearson correlation analysis was performed among the five candidate biomarkers to identify redundant features (Fig. 2d). FPDcF and FPD exhibited the highest correlation ($r = 0.95$), indicating near-identical electrophysiological information except for Fridericia's QT-correction factor. Correlations between FPDcF or FPD and the remaining three biomarkers remained below 0.30, indicating sufficient independence. To prevent multicollinearity, we retained only FPDcF as the primary repolarization biomarker, justified by its physiologically relevant heart-rate correction and established use as the standard parameter in clinical QTc assessment. The final input feature set comprised four

complementary biomarkers capturing repolarization kinetics (FPDcF), intrinsic contractile rate (beat period), cellular excitability and force (spike amplitude), and electrical instability (CoV), thereby providing comprehensive coverage of the integrated electrophysiological responses to drug perturbation and improved discrimination between TdP risk categories compared to single-biomarker approaches.

Machine learning model development for TdP risk classification

To develop an AI-driven predictive model for TdP risk classification, we employed four complementary machine learning architectures including Logistic Regression (LR), Artificial Neural Networks (ANN), Random Forest (RF), and Extreme Gradient Boosting (XGB). Each algorithm offers distinct learning paradigms suited to different data characteristics, with the aim of capturing complementary aspects of electrophysiological patterns underlying TdP risk.

All four models underwent systematic hyperparameter optimization using grid search with 5-fold stratified cross-validation on the training dataset derived from 12 CiPA reference compounds (Fig. 2c). Stratified cross-validation ensured balanced representation of risk categories across folds, preventing performance inflation from class imbalance. Optimal hyperparameters were selected based on mean cross-validated Area Under the Curve (AUC) and accuracy. The hyperparameter ranges evaluated included the following values for each model. For LR, regularization strength C was evaluated from 0.001 to 10 and penalty type was either L1 or L2. For ANN, hidden layer neurons were tested from 4 to 16, learning rate from 0.001 to 0.1, and dropout from 0.0 to 0.3. For RF, number of trees ranged from 50 to 500, maximum depth from 5 to 20, and minimum samples split from 2 to 10. For XGB, learning rate was evaluated from 0.01 to 0.3, maximum depth from 3 to 8, and L2 regularization lambda from 0 to 10. Once optimal hyperparameters were identified, each model was retrained on the full training dataset and subsequently evaluated on the independent test set derived from 16 CiPA reference drugs to assess generalization performance.

LR was employed as a linear baseline model providing probabilistic binary classification through direct biomarker coefficients. ANN with a 4-input layer, single hidden layer containing 8 neurons with ReLU activation, and sigmoid output layer were used to capture non-linear relationships between biomarkers and TdP outcome (Fuadah et al. 2023, 2024; Pramudito et al. 2024; Park et al. 2025). RF, trained as an ensemble of decision trees on random feature subsets, was implemented to detect feature interactions and threshold effects while remaining robust to outliers in MEA measurements (Fuadah et al. 2023, 2024; Pramudito et al. 2024; Park et al. 2025). XGB with sequential tree boosting and L1/L2 regularization achieved

iterative optimization of mispredicted samples while preventing overfitting on the limited dataset (Fuadah et al. 2023, 2024; Pramudito et al. 2024; Park et al. 2025). This spectrum of approaches ranging from linear (LR) to non-linear (ANN), tree-based (RF), and regularized boosting (XGB) enabled comprehensive coverage of different pattern types in the data.

Testing scenario and performance evaluation

To rigorously evaluate the generalization performance of each trained model, we implemented a repeated sampling approach with 10,000 testing iterations, following the methodology established by Li et al. 2018 (Supplementary Fig. 1a) (Li et al. 2019; Yoo et al. 2021; Jeong et al. 2022; Fuadah et al. 2023; Pramudito et al. 2024; Park et al. 2025). Rather than relying on a single test evaluation, this approach generates multiple independent test sets from the same pool of held-out compounds, thereby providing a comprehensive assessment of model robustness and performance variability across different sampling configurations. For each of the 10,000 iterations, we randomly selected one sample from each of the 16 CiPA reference compounds comprising the test set, thereby generating 10,000 unique test datasets each containing 16 independent observations (Li et al. 2019; Yoo et al. 2021; Jeong et al. 2022; Fuadah et al. 2024). Each of these 10,000 test datasets was independently evaluated against the four trained machine learning models (LR, ANN, RF, and XGB), with each model producing probabilistic predictions and class assignments for every sample in each test set.

From each testing iteration, multiple performance metrics were computed to comprehensively evaluate model classification quality. The metrics calculated included Area Under the Curve (AUC), accuracy, sensitivity (also termed true positive rate or recall), specificity (true negative rate), positive likelihood ratio (LR+), negative likelihood ratio (LR−), and F1-score. Accuracy was calculated according to Eq. 1, representing the proportion of correctly classified samples. Sensitivity was computed according to Eq. 2, wherein sensitivity represents the proportion of actual high-risk drugs correctly identified by the model. Specificity was calculated according to Eq. 3, representing the proportion of actual low-risk drugs correctly identified by the model. LR+ was derived according to Eq. 4, quantifying how much the odds of high-risk classification increase given a positive test result. LR− was calculated according to Eq. 5, quantifying how much the odds of high-risk classification decrease given a negative test result. The F1-score, computed according to Eq. 8, provides a harmonic mean of precision and recall, offering a balanced assessment of model performance particularly useful when classes are imbalanced (Li et al. 2019; Yoo et al. 2021; Jeong et al. 2022; Pramudito et al. 2024).

$$Accuracy = \frac{TP + TN}{TP + TN + FP + FN} \quad \text{Eq. 2}$$

$$Sensitivity = \frac{TP}{TP + FN} \quad \text{Eq. 3}$$

$$Specificity = \frac{TN}{TN + FP} \quad \text{Eq. 4}$$

$$LR+= \frac{Sensitivity}{1 - Specificity} \quad \text{Eq. 5}$$

$$LR-= \frac{1 - Sensitivity}{Specificity} \quad \text{Eq. 6}$$

$$Precision = \frac{TP}{TP + FP} \quad \text{Eq. 7}$$

$$F1Score = \frac{2 \times Precision \times Sensitivity}{Precision + Sensitivity} \quad \text{Eq. 8}$$

Across all 10,000 testing iterations, the distribution of each performance metric was summarized using the median value and the lower and upper bounds of the 95 percent confidence interval (95% CI), thereby providing a robust statistical summary that is resistant to outliers while capturing the range of model performance (Fuadah et al. 2023, 2024; Pramudito et al. 2024; Park et al. 2025). This repeated sampling strategy with confidence interval reporting enables comprehensive evaluation of model generalization and identifies the typical performance range across multiple independent test configurations drawn from the same pool of reference compounds. Following validation of all four individual models on the CiPA reference compounds test set, we developed a soft-voting ensemble classifier to predict TdP risk for the four non-cytotoxic anticancer agents (idarubicin, erlotinib, sunitinib, and cyclophosphamide). Given that each of the four individual classification models demonstrated robust and consistent performance across the 10,000 repeated testing iterations, with comparable AUC and accuracy metrics, we selected a soft-voting ensemble approach to leverage the complementary strengths of all four models rather than relying on a single best-performing algorithm. The soft-voting ensemble aggregates probabilistic predictions from all four base classifiers through weighted averaging of their predicted class probabilities for each

test sample.

Supplementary Figures

a

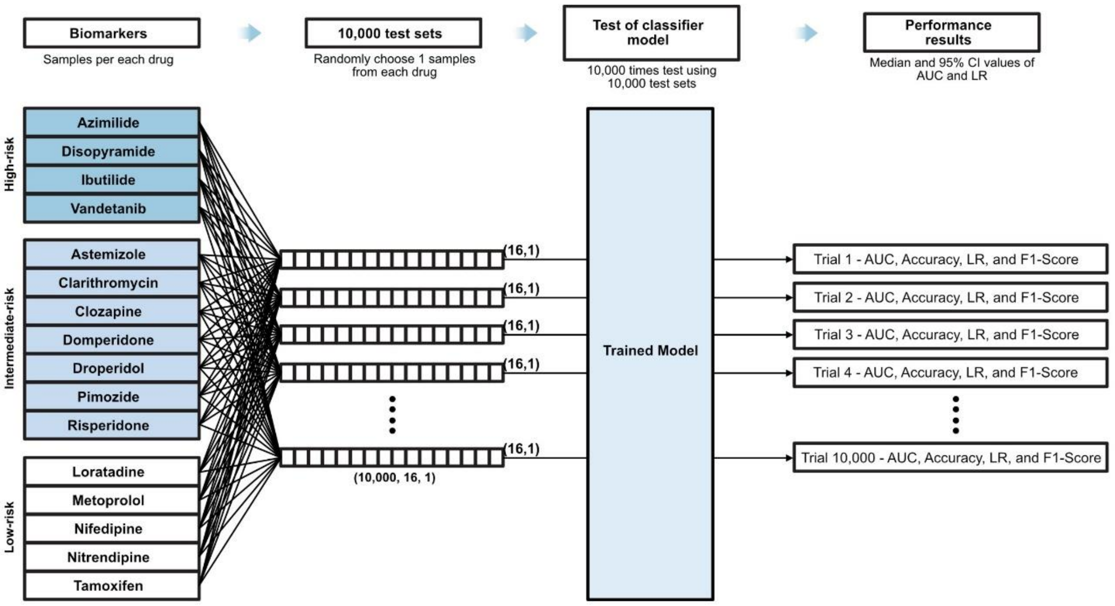

b

Scenario	Models	Accuracy	ROC-AUC	LR+	LR-	Classification Error
FPDcf, Spike Amplitude, Beat Period, Beating Irregularity	LR	0.875 (0.813, 0.938)	0.964 (0.891, 0.982)	4.545 (4.091, 5.000)	0.113 (0.000, 0.227)	0.125 (0.063, 0.188)
	ANN	0.875 (0.813, 0.938)	0.982 (0.909, 1.000)	4.545 (4.091, 5.000)	0.114 (0.000, 0.227)	0.125 (0.063, 0.188)
	RF	0.875 (0.813, 0.938)	0.964 (0.846, 0.982)	4.546 (4.091, 5.000)	0.114 (0.000, 0.227)	0.125 (0.063, 0.188)
	XGB	0.875 (0.750, 0.938)	0.964 (0.891, 0.982)	4.091 (1.515, 5.000)	0.114 (0.000, 0.227)	0.125 (0.063, 0.250)
FPD, Spike Amplitude, Beat Period, Beating Irregularity	LR	0.875 (0.813, 0.938)	0.964 (0.873, 0.982)	4.545 (4.091, 5.000)	0.114 (0.000, 0.227)	0.125 (0.063, 0.188)
	ANN	0.875 (0.813, 0.938)	0.982 (0.909, 1.000)	4.545 (4.091, 5.000)	0.114 (0.000, 0.227)	0.125 (0.063, 0.188)
	RF	0.875 (0.813, 0.938)	0.964 (0.909, 0.982)	4.546 (2.500, 5.000)	0.114 (0.000, 0.227)	0.125 (0.063, 0.188)
	XGB	0.875 (0.813, 0.938)	0.873 (0.827, 0.982)	4.545 (2.273, 5.000)	0.114 (0.000, 0.227)	0.125 (0.063, 0.188)
FPDcF	LR from Blinova		0.9412			
FPD	LR from Blinova		0.8824			
Spike Amplitude	LR from Blinova		0.5882			
Beat Period	LR from Blinova		0.8431			
Beating Irregularity	LR from Blinova		0.5490			
Previous Study	Blinova et. al. 2018 (FPDcF + LR)	-	0.872	-	-	-
	Serrano et. al. 2023 (TSM + LR)	-	0.950	-	-	-
	Yun-Gwi Park et. al. 2025 (FPDcF, FPD, Amplitude, Beat Period + ANN)	0.800 (0.670, 0.870)	0.940 (0.810, 1.000)	4.000 (2.000, 10.000)	0.000 (0.000, 0.570)	-
	Pramudito et. al. 2026 (2 features FPDcF)	-	1.000 (0.980, 1.000)	1.0E+6 (3.000, 1.0E+6)	0.000 (0.000, 0.125)	0.000 (0.000, 0.125)

Supplementary Fig.1 Workflow for AI-driven Deep Learning Architecture for TdP Risk Prediction and Performance Evaluation via ROC Curve Analysis. (**a**) Schematic of 10,000 repeated sampling validation strategy. Biomarkers from 16 CiPA reference test compounds are randomly sampled to generate 10,000 independent test sets, each containing one sample per compound. Each test set is evaluated against the trained classifier models, generating performance metrics (AUC, accuracy, sensitivity, specificity, LR+, LR−, and F1-score) for all 10,000 iterations. Performance results are summarized as median values with lower and upper 95% confidence interval bounds, providing robust statistical assessment of model generalization across multiple independent test configurations. **(b)** Detailed performance metrics from 10,000 repeated testing iterations across all scenarios and models. The table presents median performance values with lower and upper 95% confidence interval bounds for accuracy, ROC-AUC, LR+, LR−, and classification error across four machine learning models (LR, ANN, RF, XGB) under Scenario 1 (FPDcF-based) and Scenario 2 (FPD-based) biomarker combinations. Single-biomarker LR models and previous literature studies are included for comparative reference (Blinova K, Dang Q, Millard D, Smith G, Pierson J, Guo L, Brock M, Lu HR, Kraushaar U, Zeng H, Shi H, Zhang X, Sawada K, Osada T, Kanda Y, Sekino Y, Pang L, Feaster TK, Kettenhofen R, Stockbridge N, Strauss DG 2018; Serrano et al. 2023; Park et al. 2025)

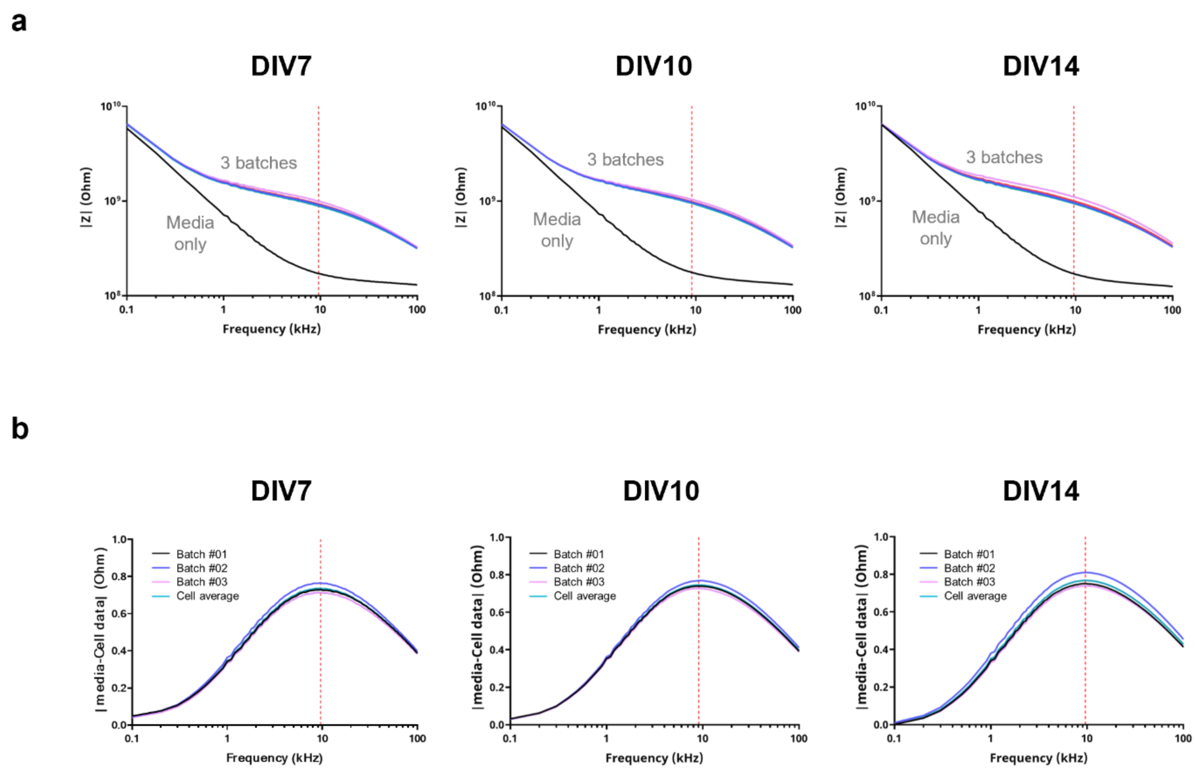

Supplementary Fig. 2 Screening of hiPSC-CM specific impedance frequency in AtlaZ platform. **(a)** Representative impedance trace obtained using the AtlaZ system from media-only wells (black line) and hiPSC-CMs (Cardiosight®-S) from three independent bathes cultured for 7, 10, and 14 days. Impedance measurements were recorded across a frequency range of 0.1-100 kHz. **(b)** Gap analysis of impedance measurements across frequencies. The gap value was defined as the absolute difference between impedance measured in media-only wells and impedance measured in cell-containing wells at the same frequency. Data from three independent hiPSC-CMs (Cardiosight®-S) batches are shown along with the mean gap value. The red dashed line indicates the frequency at which the maximum gap was observed, which was selected the optimal frequency for subsequent impedance analyses (9.6 kHz)

References

Blinova K, Dang Q, Millard D, Smith G, Pierson J, Guo L, Brock M, Lu HR, Kraushaar U, Zeng H, Shi H, Zhang X, Sawada K, Osada T, Kanda Y, Sekino Y, Pang L, Feaster TK, Kettenhofen R, Stockbridge N, Strauss DG GG (2018) International Multisite Study of Human-Induced Pluripotent Stem Cell-Derived Cardiomyocytes for Drug Proarrhythmic Potential Assessment. Cell Rep 24:3582–3592. https://doi.org/10.1016/j.celrep.2018.08.079.International

Fuadah YN, Qauli AI, Marcellinus A, et al (2023) Machine learning approach to evaluate TdP risk of drugs using cardiac electrophysiological model including inter-individual variability. Front Physiol 14:1–18. https://doi.org/10.3389/fphys.2023.1266084

Fuadah YN, Qauli AI, Pramudito MA, et al (2024) A stacking ensemble machine learning model for evaluating cardiac toxicity of drugs based on in silico biomarkers. CPT Pharmacometrics Syst Pharmacol 13:2159–2170. https://doi.org/10.1002/psp4.13229

Jeong DU, Yoo Y, Marcellinus A, et al (2022) Proarrhythmic risk assessment of drugs by dVm/dt shapes using the convolutional neural network. CPT Pharmacometrics Syst Pharmacol 11:653–664. https://doi.org/10.1002/psp4.12803

Li Z, Ridder BJ, Han X, et al (2019) Assessment of an In Silico Mechanistic Model for Proarrhythmia Risk Prediction Under the CiPA Initiative. Clin Pharmacol Ther 105:466–475. https://doi.org/10.1002/cpt.1184

Park YG, Park NK, Lee Y, et al (2025) A machine learning platform for genotype-specific cardiotoxicity risk prediction using patient-derived iPSC-CMs. J Adv Res. https://doi.org/10.1016/j.jare.2025.07.030

Pramudito MA, Fuadah YN, Qauli AI, et al (2024) Explainable artificial intelligence (XAI) to find optimal in-silico biomarkers for cardiac drug toxicity evaluation. Sci Rep 14:1–24. https://doi.org/10.1038/s41598-024-71169-w

Serrano R, Feyen DAM, Bruyneel AAN, et al (2023) A deep learning platform to assess drug proarrhythmia risk. Cell Stem Cell 30:86-95.e4. https://doi.org/10.1016/j.stem.2022.12.002

Yoo Y, Marcellinus A, Jeong DU, et al (2021) Assessment of Drug Proarrhythmicity Using Artificial Neural Networks With in silico Deterministic Model Outputs. Front Physiol 12:1–7. https://doi.org/10.3389/fphys.2021.761691
